# Phosphorus applications adjusted to optimal crop yields can help sustain global phosphorus reserves

**DOI:** 10.1038/s43016-024-00952-9

**Published:** 2024-03-25

**Authors:** R. W. McDowell, P. Pletnyakov, P. M. Haygarth

**Affiliations:** 1 Faculty of Agriculture and Life Sciences, Lincoln University, Lincoln, New Zealand; 2 AgResearch, Lincoln Science Centre, Christchurch, New Zealand; 3 Lancaster Environment Centre, Lancaster University, Lancaster, UK

**Keywords:** Agriculture, Geography, Element cycles, Environmental impact

## Abstract

With the longevity of phosphorus reserves uncertain, distributing phosphorus to meet food production needs is a global challenge. Here we match plant-available soil Olsen phosphorus concentrations to thresholds for optimal productivity of improved grassland and 28 of the world’s most widely grown and valuable crops. We find more land (73%) below optimal production thresholds than above. We calculate that an initial capital application of 56,954 kt could boost soil Olsen phosphorus to their threshold concentrations and that 28,067 kt yr^−1^ (17,500 kt yr^−1^ to cropland) could maintain these thresholds. Without additional reserves becoming available, it would take 454 years at the current rate of application (20,500 kt yr^−1^) to exhaust estimated reserves (2020 value), compared with 531 years at our estimated maintenance rate and 469 years if phosphorus deficits were alleviated. More judicious use of phosphorus fertilizers to account for soil Olsen phosphorus can help achieve optimal production without accelerating the depletion of phosphorus reserves.

## Abstract (web summary)

Ongoing depletion rates of phosphorus reserves might pose a challenge to future food security. This Analysis estimates the effects of matching plant-available soil Olsen P concentrations with thresholds for optimal yields of grassland and 28 crops on the longevity of global P reserves.

## Main

Human existence over the past century has depended on the production of phosphorus fertilizer and its application to agricultural soils to drive food production^1^. Phosphorus fertilizer production relies on geologic rock phosphorus supplies extracted from mines at relatively few locations and requires transportation and distribution before application to farmlands worldwide. The global population is projected to increase to nearly 10 billion people by 2050^2^. It has been projected that feeding this increased population will require an additional 500 million hectares of arable land unless phosphorus can be more efficiently used to boost or maintain optimal crop yields^3^. Most of this efficiency will be created by local management solutions that apply phosphorus fertilizers only where they are needed and by making better use of available soil phosphorus concentrations^4^.

To boost crop yields, we must close the gap between actual and potential yields with more judicious application of fertilizer to match available soil phosphorus concentrations and crop demands^5^. Global estimates put the overapplication of phosphorus fertilizers at 30–40% relative to crop and grassland requirements^5–7^. Some of this can be redistributed, but the efficiency gain may still not meet crop and food demands^8^. Redistribution and the lowering of soil phosphorus concentrations, especially in some jurisdictions such as China and Europe, will also help avoid the risk of surface water quality deterioriation^9^. However, the spatial distribution of soil phosphorus concentrations is uncertain. Previous work has modelled the spatial distribution of available soil phosphorus concentrations and stocks in Africa and Europe^10,11^. Estimates of concentrations and stocks have also been made at a global level, but these are of total phosphorus, not plant-available phosphorus in agricultural soils^12,13^. Additional estimates of phosphorus flows have been derived by mass balance models that consider factors such as plant uptake, weathering and global lithology data^3,14–17^, but again these do not estimate plant-available phosphorus.

Accurate knowledge of where crops are grown and the available soil phosphorus concentration of those soils is a key step in reducing yield gaps and making optimal use of phosphorus fertilizer reserves. Recent work has updated and improved the spatial resolution of the major crops, rangeland, improved grassland (for example, for intensive forage production) and forest land for the period 2005–2015^18,19^. Additional data are now also available for available soil phosphorus (as Olsen extractable phosphorus) for 2001–2019 (with most data available on average for 2009)^20^. We therefore aimed to link available soil phosphorus concentrations to thresholds established for optimal crop growth. This enables us to better match phosphorus fertilizer supply to crop demand. Note that in focusing on phosphorus, this analysis did not consider whether other climatic or biophysical factors were limiting production. Our second aim was to determine the effect that alleviating a deficit, maintaining a threshold for optimal crop yield and redistributing excess phosphorus applications would have on phosphorus reserves. Controversial concerns have been raised that phosphorus fertilizer supplies could become constrained or exhausted in 30 to 300 years^21,22^. While we recognize that such supplies refer only to easily mineable phosphorus reserves and that constraints could also be alleviated by an increase in phosphorus supply as more reserves become economically mineable or indeed are discovered^23^, we examined how different crop phosphorus requirements compare to reserves. These data will guide future fertilizer deployment and contribute to closing yield gaps and preventing future phosphorus resource exhaustion.

## Results

### Global agronomic phosphorus requirements

**Fig. 1:**
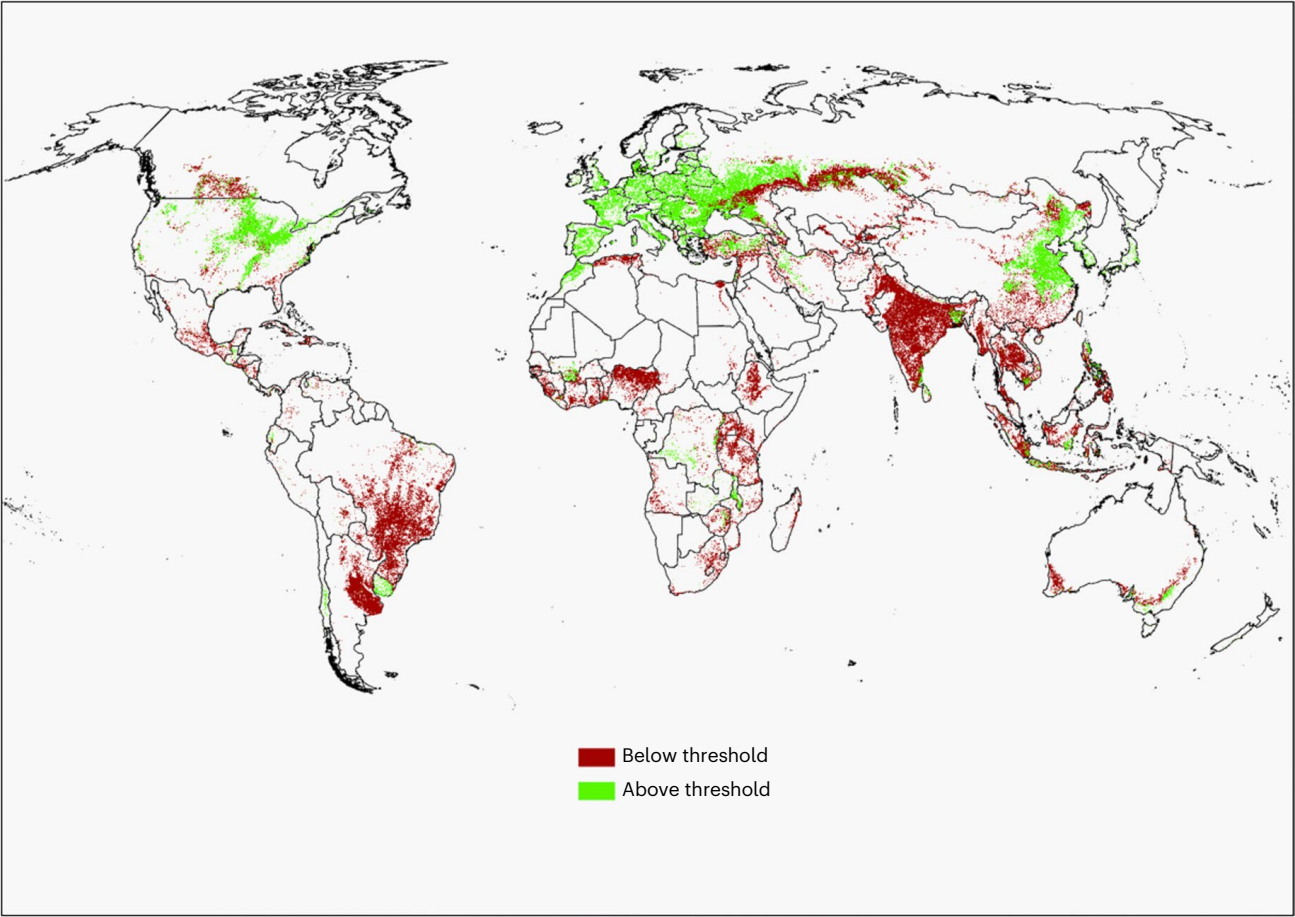
The global distribution of land area planted with rice, soybeans, maize, wheat, rye, barley, oranges or apples above or below their agronomic threshold of 15 mg kg^−1^ required for optimum production. Areas in Europe and North America above and below the threshold relate to the production of wheat and maize. Basemap from GADM (https://gadm.org/data.html) (to find how much a location is above or below 15 mg kg^−1^, see the interactive map of soil Olsen phosphorus concentrations at https://world-olsen.agr.nz/).

Maintaining topsoil Olsen phosphorus concentrations at an agronomic optimum would ensure that yields are not limited by soil phosphorus availability. Taking a global view, we estimated the land areas above (including those within 1 mg kg^−1^ Olsen phosphorus) and below the thresholds corresponding to likely agronomic optimal yield for 28 of the world’s most widely grown and valuable crop species, plus improved pasture (for intensive grazing by livestock). See Fig. 1 for an example of the spatial distribution of soil phosphorus concentrations above and below the threshold for optimal yield (15 mg kg^−1^) for rice, soybeans, maize, wheat, rye, barley, oranges or apples. We did not include forestlands, non-productive lands or rangelands in these calculations because these receive little or no phosphorus fertilizer^24^.

**Extended Data Fig. 1:**
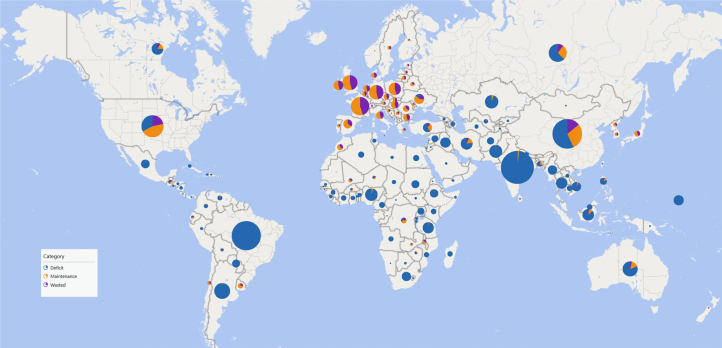
Categorical phosphorus fertiliser applications. Phosphorus fertiliser applications rates required to alleviate (deficit), maintain (maintenance) or revert (wasted) to thresholds for optimal crop production at the country level. Note that the size of the pie chart indicates the relative magnitude of required applications. Basemap from Open StreetMap (https://www.openstreetmap.org).

Globally, the proportion of cropland and improved grassland area above the agronomic thresholds required for optimal yield (27%, with a range of 16–44%) was less than that below these thresholds (73%). The continents with the largest total areas with excess phosphorus were Europe and North America, with the countries of France and the United States accounting for the highest respective proportions (Table 1 and Extended Data Fig. 1). The greatest phosphorus deficiency was observed in Asia, with India accounting the greatest proportion by country (Table 1 and Fig. 1). Between land uses, croplands were more likely to be above their respective thresholds (40%, with a range of 26–55%) than improved grasslands (29%) (Table 1).

**Table 1:** Continental and global land areas either above or below the thresholds for soil Olsen phosphorus concentrations relating to the optimal yield of 28 crops and improved grassland

Continent	Position relative to the threshold	Cropland			Improved grassland		
		Area (km^2^)	Percentage area (%)	90% confidence interval range for percentage area	Area (km^2^)	Percentage area (%)	90% confidence interval range for percentage area
Africa	Above	477,888	31	13–46	24,942	9	7–11
	Below	1,066,772	69	54–87	266,809	91	89–93
Asia	Above	1,324,088	30	18–46	177,654	16	10–40
	Below	3,056,479	70	54–82	936,695	84	60–90
Europe	Above	1,274,218	88	76–98	381,974	73	68–87
	Below	167,502	12	2–24	140,071	27	13–32
North America	Above	725,274	63	34–83	166,626	27	12–61
	Below	429,125	27	17–66	450,367	73	39–88
Oceania	Above	40,452	27	10–64	107,226	34	9–51
	Below	109,069	73	36–90	206,807	66	41–91
South America	Above	71,218	6	3–17	67,482	24	16–26
	Below	1,046,024	94	83–97	212,340	76	74–84
Global sum	Above	3,913,138	40	26–55	925,904	29	20–49
	Below	5,874,971	60	45–74	2,213,089	71	51–80

The soil Olsen phosphorus concentrations at each crop location are given as 90% confidence intervals. Note that areas above the threshold include those soils within 1 mg kg^−1^ of the Olsen phosphorus threshold. The thresholds of the Olsen phosphorus concentrations for the different land uses are 20 mg kg^−1^ for improved grasslands and between 5 and 60 mg kg^−1^ for croplands, depending on the crop (including cereals, root crops and fruits).

**Fig. 2:**
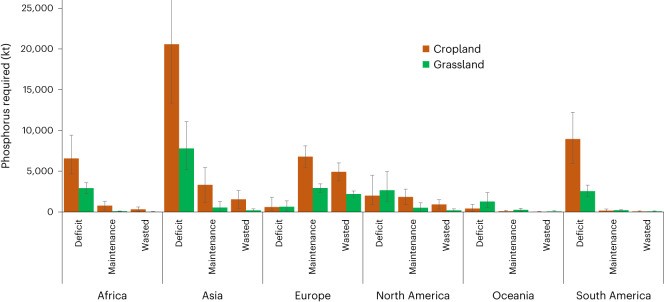
Continental phosphorus amounts required to reach (deficit), maintain (maintenance) or revert to (wasted) thresholds of Olsen phosphorus relating to optimal yield for 28 crops and improved grassland. The bars show the mean soil Olsen phosphorus concentration, and the error bars represent the 90% confidence interval. The maintenance phosphorus (in kt yr^−1^) is the amount of phosphorus applied annually to maintain the soil Olsen phosphorus concentration at the agronomically optimal threshold for each crop. The deficit phosphorus (in kt applied once or spread over multiple applications) is the amount required as capital application in one year to reach the agronomically optimal Olsen phosphorus concentration threshold. The wasted phosphorus (in kt yr^−1^) is the difference in the amount applied annually to maintain the Olsen phosphorus level above versus at the agronomically optimal threshold.

We next calculated how much phosphorus should be applied in one year (often called a ‘capital’ application) to alleviate phosphorus deficits and increase the current Olsen phosphorus concentration to the agronomic threshold for each crop. We termed this ‘deficit phosphorus’ (Fig. 2). We also calculated maintenance phosphorus as the phosphorus required to maintain a threshold in soil Olsen phosphorus concentration, as well as the difference in the phosphorus required to maintain the current Olsen phosphorus concentration and that required to maintain the threshold concentration. We called this difference ‘wasted phosphorus’ as it represents phosphorus that does not need to be applied. We validated our calculations for maintenance phosphorus applied using data for 24 countries (Supplementary Information).

Globally, the phosphorus required as a capital (one-off) application to alleviate a deficit was estimated to be 56,954 kt (ranging from 36,459 to 86,329 kt); much more (39,109 kt, ranging from 25,150 to 59,685 kt) was required for cropland than for improved pasture. The greatest phosphorus deficit was observed in cropland, largely in Asia (Fig. 2, Supplementary Table 2 and Extended Data Fig. 1).

The global maintenance phosphorus requirement was calculated to be 17,500 kt, dominated by cropland in Europe and Asia. If only considering cropland, the sum of maintenance and wasted phosphorus was 20,774 kt (ranging from 14,249 to 29,174 kt); if also including improved grassland, the sum was 28,067 kt (ranging from 19,672 to 39,549 kt). This means that wasted phosphorus was calculated to be 10,556 kt, again dominated by Europe and Asia, and was accounted for by wheat and improved grassland in Europe and maize and rice in Asia (Extended Data Fig. 1).

Globally, 47,000 kt of P_2_O_5_ or 20,500 kt of phosphorus is applied to agricultural soils each year as fertilizer^25^. No data were available at the 1 km^2^ scale to indicate where and to which land uses the current global production of phosphorus fertilizers is applied. However, the close agreement between our estimates of maintenance requirements (especially for cropland) and application rates suggests that on a global level our calculations are robust.

### Implications for phosphorus reserves

As of 2020, the global estimated stock of phosphate rock reserves amounted to 71,000,000 kt, which equates to 9,301,310 kt of phosphorus after accounting for the P_2_O_5_ concentration in phosphate rock (30%) and the conversion of P_2_O_5_ into phosphorus^25^. At the present rate of application (20,500 kt yr^−1^), the currently estimated 9,301,310 kt of phosphorus reserves would be exhausted in 454 years if used only to grow crops. We compared this exhaustion rate to rates derived if we substituted our estimate of maintenance and an optimal scenario that corrected any deficit before applying maintenance. Under our estimated maintenance rates, phosphorus reserves would be exhausted in 531 years (cropland and improved pasture, with a range of 373–766 years). However, if the phosphorus that is currently overapplied (that is, waste phosphorus) is slowly redistributed to areas of deficit and maintenance is applied, this optimal scenario would see phosphorus reserves exhausted in 469 years (with a range of 357–578 years).

## Discussion

Estimates have been made in the past of global phosphorus fertilizer requirements to meet crop demand^3,7^. These have been calculated by combining fertilizer use data at the country scale with point-scale (for example, at a 0.5-degree resolution) estimates of livestock numbers and manure production rates^26^ and crop types, locations and application rates^27^. The authors of these estimates concluded that insufficient phosphorus fertilizer is being applied to meet targets for crop and food demand. A large area of uncertainty in these estimates was the phosphorus supplied from the soil^16^. To estimate phosphorus supply from the soil, inputs (for example, fertilizers and manures) and outputs (for example, erosion, leaching and crop offtakes) were combined with spatial estimates of available phosphorus concentrations before fertilizer applications (that is, in virgin soils)^3,6,14^. While this can create finely resolved spatial data, we argue that estimates of available soil phosphorus are more reliably derived from observations^20^ and detailed data on soil chemical composition (for example, phosphorus retention) to account for soil specific variation^28^. These data allow us to better match phosphorus supply to crop demand and fertilizer requirements by soil type^29^. For instance, much more phosphorus is required to achieve optimal crop yield in high-phosphorus-fixing soils in the tropics than for lower-phosphorus-fixing soils in temperate regions^30^.

Published estimates of the proportion of land in soil phosphorus deficit are 30–32% for cropland^7^ and 43% for grassland^6^. Mogollón et al.^3^ argued that by alleviating this deficit we could produce sufficient food to feed the global population in 2050 and avoid expanding cropland area. Sattari et al.^16^ estimated the phosphorus fertilizer required between 2008 and 2050 to alleviate the deficit at 1,070,000–1,200,000 kt. Focusing on cropland, our data suggest that an application to alleviate the deficit, coupled with 42 years of maintenance phosphorus fertilizer, equates to 911,617 kt, within 10% of their lower estimate. Including improved grassland brings our estimate to 1,235,768 kt (<3% different). The estimates of Mogollón et al.^3^ and Sattari et al.^16^ included phosphorus inputs from fertilizers and manures, but not recycling from municipal wastewater, which could supply about 4% of crop demands^31^. Since available soil phosphorus is influenced by either form of input, our estimates are comparable.

Our estimates contain three main sources of uncertainty: the modelling of observations used as input data to our estimates, the spatial resolution (and whether it is fine enough to represent land management practices) and the use of a single threshold for crops. Concerning the first, we present confidence intervals for soil phosphorus concentrations that have been used in all subsequent calculations. We also calculated the mean coefficient of variation for the residuals (the difference between observed and modelled values) for soil Olsen phosphorus concentrations. This was low at 6.9% but did vary, especially for points >25 mg kg^−1^ Olsen phosphorus (Supplementary Table 2). The mean of absolute residuals in the range where most crops had their threshold for optimal yield (10–25 mg kg^−1^) was even lower (1.4%).

Although we have confidence in the matching of modelled and observed data on a point basis, the data suffer from poor representativeness of practices in sparsely sampled regions. For instance, the mean distance in the point data used as inputs for our estimates is 1,267 km^2^. If the ~33,000 data points were evenly distributed across land used for agricultural production (47.4 million km^2^), the spatial density would be 1,436 km^2^. The density of sampling was skewed towards Europe and North America (50% and 29% of sampled points, respectively). However, these are the continents with the largest areas of intensive agriculture. This maximizes the likelihood of capturing shifts in soil Olsen phosphorus caused by intensification (for example, an expansion of confined animal feeding operations in North America^32^) but would still miss changes that occur at a smaller scale.

Finally, we used a single threshold for crops, rounding those ±3 mg kg^−1^ Olsen phosphorus to the nearest interval of 5 mg kg^−1^. We may have misclassified thresholds for crops that have a legitimate reason to warrant a lower or higher threshold (for example, a local combination of soil type, slope and climate). These errors would be greatest for the crops with the largest areas. In assessing the validity of maintenance applications in 24 countries (Supplementary Information), the amounts applied were close to those recorded for four widely grown crops (maize, rice, soybeans and wheat). This gives us confidence that the thresholds were sensible at least for those crops in those countries. We do note that thresholds can be strongly influenced by soil phosphorus retention (for example, oxisols in Brazil), and we have therefore included a dynamic adjustment to maintain a threshold according to soil phosphorus retention.

### Implications for phosphorus reserves

Estimates of the effect of phosphorus fertilizer application rates on the longevity of phosphorus reserves have been controversial, with predictions about when phosphorus reserves would be exhausted ranging from a few decades to hundreds of years hence^33,34^. All agree that phosphate reserves are finite but disagree on the size and definition of phosphorus reserves^23,35^. We have used an independent and generally well-accepted source (US Geological Survey)^25^ and have estimated that through a combination of alleviating deficits by avoiding overapplication in areas of surplus and then applying no more than maintenance rates, current reserves would be exhausted in 469 years (ranging from 357 to 578 years). Estimates of reserves are seldom downgraded as better technology makes more phosphorus available. There is also pressure on phosphorus to be conserved by increasing our intake of plant-based foods instead of using phosphorus to feed crops for livestock that are then used to feed people—wasting some phosphorus in unutilized fodder or animal products^36^. Additional pressure via policy to improve water quality make it unlikely that deficits in improved grasslands for intensive land uses such as dairying will be alleviated^37^. Our conclusion from these data and trends is that through adjusting phosphorus fertilizer application rates, we can achieve optimal yields without increasing the rate at which phosphorus reserves are being depleted.

### Improved phosphorus use efficiency

Efficiencies gained through the better use of phosphorus for production will probably be influenced by a balance between inputs and outputs associated with the recycling of phosphorus in manure and losses to water, respectively. This balance will be influenced by climate change, which will shift where crops are grown^38^ and probably increase phosphorus losses to water owing to an increase in the number of large storms^39^. Globally, annual manure inputs of 1,600 to 7,200 kt (ref. ^14^) are in the same range as losses to water (2,100 to 6,700 kt annually)^17,40^. Although much less than fertilizer application rates, if not distributed widely these inputs could result in regional hotspots of soil phosphorus concentrations and loss^32^ and affect the need for phosphorus fertilizer. Data are becoming available for the movement of manure phosphorus^41^ but are currently restricted to the continental United States.

The relationship between crop yield and soil phosphorus concentrations is variable, affected by factors such as the availability of other nutrients, soil types and climate. We altered our estimated application rates to account for variable soil types according to phosphate retention. However, increasing evidence is becoming available to look at climate and soil chemical characteristics. For example, Ros et al.^29^ collated a database of 67 studies and 1,227 observations of grassland yield and soil phosphorus concentrations and were able to derive optimal yield thresholds that respond to soil pH and location (temperate versus tropical). Although more refined estimates are available for some important crops such as wheat in some regions^42^, underinvestment in generating the equivalent data for most crops means that there is still room for improvement in crop phosphorus use efficiency.

Recent data have also been produced on the influence of climate change on crop yields. The consensus is that increasing temperatures are likely to reduce the yields of many crops^43^ and livestock production^44^. However, increasing temperatures coupled with changes in the plant availability of soil phosphorus^45^ could be used to modify sowing dates and improve yields^46^. Combining climate change and crop growth models, Jägermeyr et al.^47^ estimated the effect on global yield, showing a net decrease (the midpoint for the SSP585 scenario) of 15% for maize and 2% for soybean and a net increase of 14% for wheat and 2% for rice by 2099. Clearly, these yield changes would affect phosphorus requirements. Indeed, these percentages translate to a decrease in annual maintenance phosphorus for maize and soybean of 561 kt and an increase of 1,409 kt for wheat and rice (Supplementary Information), which when combined suggest that phosphorus demand may not increase much (848 kt or 4% of 2020 application rates). It is also likely that other crops may show decreases and increases, but no data were available to discern whether they would be material to phosphorus requirements and hence phosphorus reserves.

Going beyond matching crop requirements to soil phosphorus concentrations, better crop yields could be achieved by improving phosphorus use efficiency in soils and crops. Increasing phosphorus use efficiency includes making unavailable (that is, non-Olsen-extractable) phosphorus available to plants. The fundamental mechanisms of how non-Olsen-extractable phosphorus could be made plant available remain unclear and should certainly be a priority for future related studies. Syers et al.^48^ have estimated that as much as 80% of total soil phosphorus could be accessible through, for example, improvements in plant genetics. Globally, the phosphorus use efficiency of wheat, barley, soybean and rice yields decreased in the 1960s, only recovering to similar levels over the past 30 years^49^. Part of this variation in phosphorus use efficiency has been farming systems benefiting from long-term phosphorus application, resulting in domesticated crop plants that use phosphorus inefficiently^50^. Improvements in phosphorus use efficiency are likely to stem from genetic research and breeding to develop plants with rhizospheres that can explore a larger soil area and liberate soil organic or residual inorganic phosphorus via exuded organic acids and enzymes^16^. In addition to phosphorus root acquisition strategies, there will be a need to develop traits that improve future plant resistance to variations in soil moisture and weather conditions exacerbated by a changing climate^51^ as well as a need to focus on localized uptake and adoption of the solutions.

## Methods

We used modelled projections and their estimated 90% confidence intervals^20^ of mean global topsoil (0–20 cm) plant-available phosphorus concentrations (termed Olsen phosphorus^52^) at 1 km^2^ resolution to isolate areas of the globe that were above or below thresholds of Olsen phosphorus for the optimal yield of 28 crops. We used these data to provide estimates of annual phosphorus fertilizer required to maintain the threshold concentration, remove any deficit to meet the threshold and maintain a concentration above the threshold—which, by difference from maintenance, is termed wasted phosphorus. Livestock production and the movement of manure phosphorus across jurisdictions is not accounted for in our calculations. However, livestock production is accounted for through their consumption of crops or of forage produced in improved grasslands.

### Global land area with adequate phosphorus for crop growth

We surveyed the literature to determine the thresholds of Olsen phosphorus concentration that would ensure optimal growth of the 28 crops with the highest global yields and values, which together accounted for the majority of food that is consumed or traded globally^27,53^. We considered livestock and livestock products to be derived from these crops (for example, improved grasslands, maize or soybeans) and hence did not account for them separately. Although it is likely that the application of manure will cause a redistribution of phosphorus across land uses that will alter phosphorus requirements^8^, this takes time. Our analysis was a snapshot in time where the effect of redistribution over one year was thought to be minimal. We did not calculate thresholds for forestlands or rangelands as globally little to no phosphorus is applied to these land uses^24^.

**Extended Data Fig. 2:**
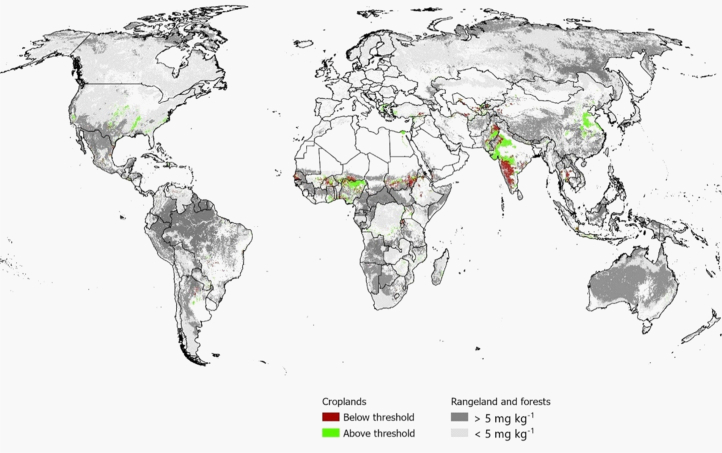
Phosphorus thresholds (5 mg kg^−1^). Map showing the land areas of either above or below the agronomic threshold of 5 mg kg^−1^ required for optimum production of cassava, millet, cotton, or sorghum. For context, areas and the Olsen phosphorus concentrations for forestland or rangeland are noted in grey with a nominal threshold of 5 mg kg^−1^^44,45^. Note that the areas in rangelands and forestlands are not included in the calculation of the maintenance, deficit or wasted phosphorus amounts.

**Extended Data Fig. 3:**
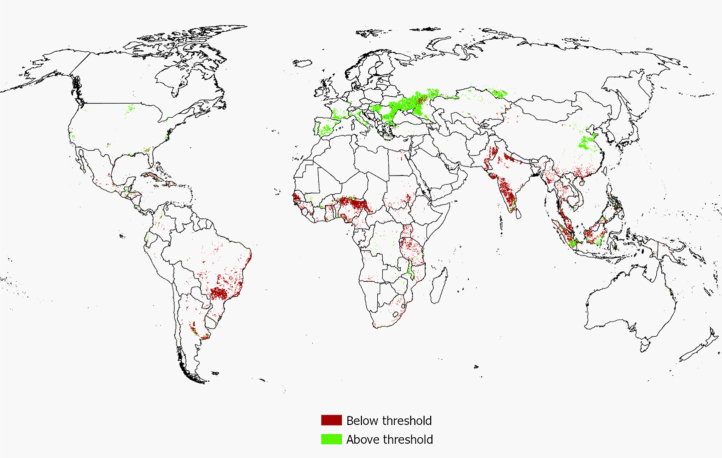
Phosphorus thresholds (10 mg kg^−1^). Map showing the land areas of either above or below the agronomic threshold of 10 mg kg^−1^ required for optimum production of groundnuts, oil palm, sunflowers, sugarcane, wine grapes, or bananas. Areas above the threshold in Europe relate largely to the production of sunflowers and wine grapes.

**Extended Data Fig. 4:**
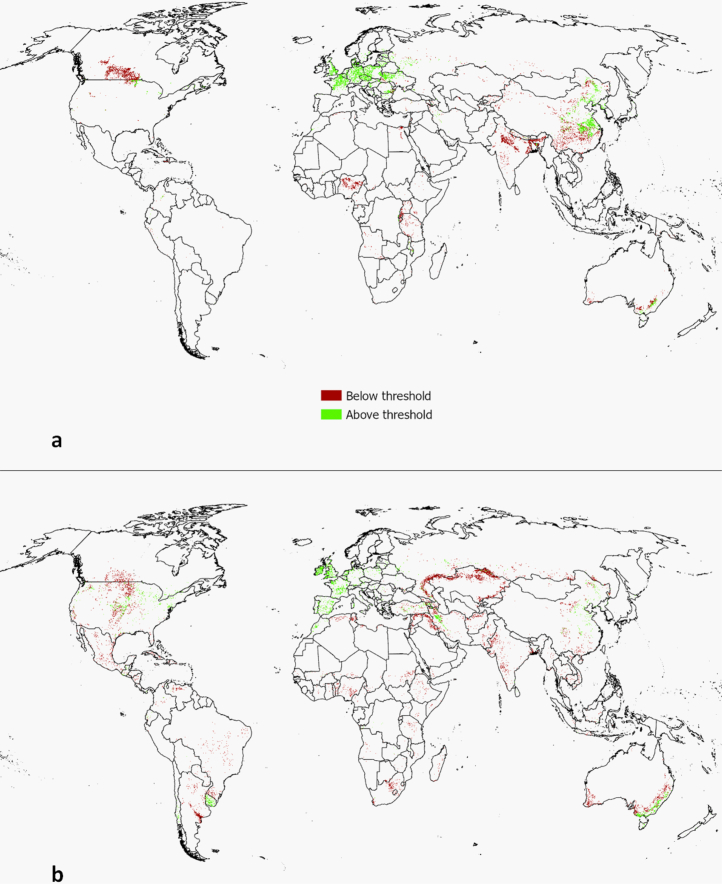
Phosphorus thresholds (20 mg kg^−1^). Map showing the land areas of either above or below the agronomic threshold of 20 mg kg^−1^ for optimum production of (a) potatoes, sugar beets, and rapeseed, or (b) improved pasture.

**Extended Data Fig. 5:**
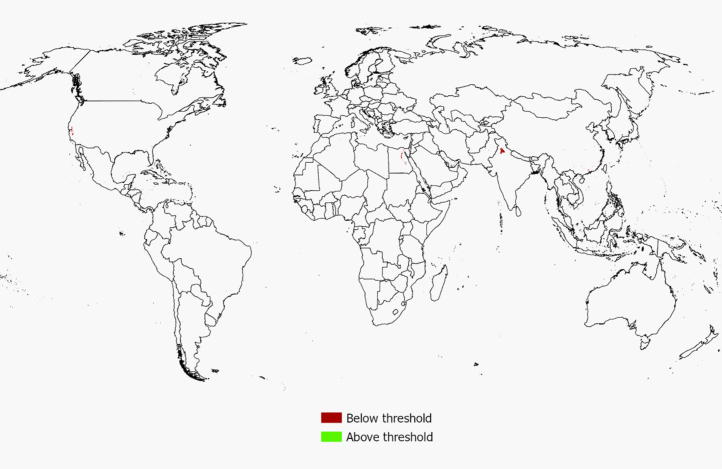
Phosphorus thresholds (40 mg kg^–1^). Map showing the land areas of either above or below the agronomic threshold of 40 mg kg^−1^ required for optimum production of cabbage, onions, or tomatoes. Areas planted in these crops are generally too small to show on a global or continental basis.

**Extended Data Fig. 6:**
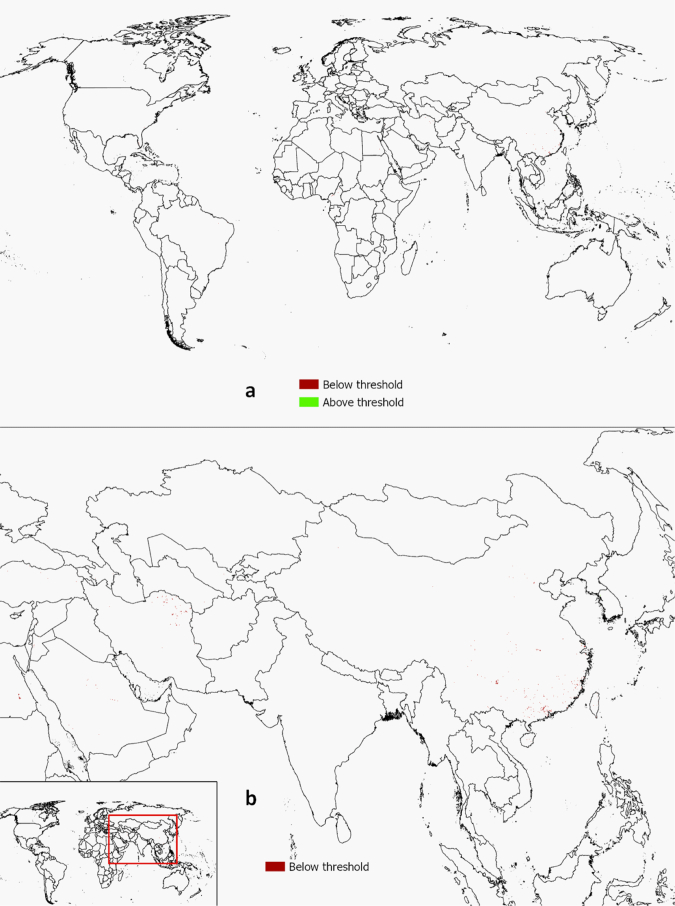
Phosphorus thresholds (60 mg kg^–1^). Map showing the land areas of either above or below the agronomic threshold of 60 mg kg^−1^ for optimum production of global (**a**) and regional (**b**) cucumbers or watermelons.

Some variation in the threshold Olsen phosphorus concentration was noted within and across crop types associated with variation in factors such as soil type, climate and treatment design (Supplementary Table 1). We therefore chose the midpoint of the concentration range for a given crop to the nearest 5 mg kg^−1^ and grouped crops according to similar midpoint values (±3 mg kg^−1^). We chose intervals of 5–20 mg kg^−1^ on the basis of industry guidelines^54,55^. The threshold soil Olsen phosphorus concentrations were grouped as follows:

- 5 mg kg^−1^ for cassava, millet, cotton and sorghum (Extended Data Fig. 2)
- 10 mg kg^−1^ for groundnut, oil palm, sunflower, sugarcane, wine grapes and banana (Extended Data Fig. 3)
- 15 mg kg^−1^ for rice, soybean, maize, wheat, rye, barley, orange and apple (Fig. 1)
- 20 mg kg^−1^ for potato, sugar beet, improved grassland and rapeseed (Extended Data Fig. 4)
- 40 mg kg^−1^ for cabbage, onion and tomato (Extended Data Fig. 5)
- 60 mg kg^−1^ for cucumber and watermelon (Extended Data Fig. 6)

To calculate the land areas below, at and above the various threshold soil Olsen phosphorus concentrations, we combined a range of geographic databases. Databases were included when they identified unique land uses. However, owing to the different spatial resolutions and degrees of accuracy, these data were applied in the following order:

1. Rangeland was classified according to the Food and Agriculture Organization rangeland class for livestock^26^.
2. Forestland, defined as evergreen- or deciduous-broadleaf-tree-covered areas, was identified on the basis of data from the European Space Agency^19^.
3. Improved grassland was classified on the basis of the European Space Agency grassland class^19^ if indicated as cropland in the National Aeronautics and Space Administration (NASA) 2010 World Cropland database^56^, which includes improved grasslands but not rangelands.
4. All other crops were distributed within the NASA cropland class according to the spatial distributions described by Monfreda et al.^27^ as updated by Grogan et al.^18^.
5. Non-productive land was categorized as all other land.

Food and Agriculture Organization and European Space Agency data were mapped at a resolution of at least 1 km^2^. NASA data were mapped at a resolution of 30 m^2^. The data of Monfreda et al.^27^ and Grogan et al.^18^ had resolutions of 100 km^2^ and 9 km^2^, respectively. Land parcels were ascribed to the most likely land cover type within the most spatially refined class. We used an area-weighted average of soil Olsen phosphorus concentrations within that class to determine if it was below, at or above the corresponding threshold. We report the land areas below, at and above the various crop thresholds at the country and continent scales.

### Annual phosphorus requirements

We calculated the annual phosphorus fertilizer amount required to maintain the threshold Olsen phosphorus concentration (‘maintenance rate’) for each crop in areas identified as being at or above the threshold concentrations. Fertilizer application rates were set to the maintenance rate for the crop (and location) if the threshold concentration was exceeded. We also calculated the phosphorus fertilizer amount required to increase the soil Olsen phosphorus concentration so that it matched the threshold concentration—often referred to as a capital application.

Both the maintenance and capital phosphorus fertilizer application rates increase with soil phosphorus sorption capacity and strength^57–62^. We therefore intersected a global map of phosphorus retention (an estimate of phosphorus sorption capacity and strength)^28^ with our maps of soil Olsen phosphorus concentrations and crop locations to estimate areas with soil Olsen phosphorus concentrations below, at and above the threshold concentrations for each crop. The various phosphorus retention classes defined by Batjes^28^ (low, moderate, high and very high) were assigned the capital phosphorus fertilizer requirements (kg ha^−1^) needed to increase the Olsen phosphorus concentration by 1 mg kg^−1^, corresponding to 6, 8, 10 and 13 kg ha^−1^, respectively, and verified in studies from Africa^59^, Asia^58^, Europe^60^ and Oceania^57,61^. To calculate the phosphorus required to maintain an Olsen phosphorus concentration, we used published equations generated to relate phosphate retention classes to the closely related buffering index^63^ and maintenance fertilizer phosphorus requirements (kg ha^−1^ yr^−1^) for a wide range of soil types in Australia^62^. The equations for maintenance fertilizer phosphorus requirements (kg ha^−1^ yr^−1^) in different phosphorus retention classes were as follows: low, 0.887 × Olsen phosphorus (mg kg^−1^), intercept = 2.84; moderate, as for low but intercept = 3.59; high, intercept = 5.59; and very high, intercept = 8.37. These phosphorus retention classes corresponded to phosphate buffering indices of <100, 100–200, 201–400 and >400, respectively. The prediction of capital and maintenance phosphorus requirements by phosphorus retention class and phosphate buffering index has been independently verified in field studies in New Zealand for phosphate retention classes ranging from low (5%) to very high (95%)^57,64^, and noted as the controlling factor for yield in high to very high phosphate retention classes in Brazil^30^. We calculated maintenance requirements for all area in crops and improved grassland. Should the current Olsen phosphorus concentration exceed the threshold, we calculated the difference in fertilizer required to maintain the current and threshold concentrations and termed this wasted phosphorus.

Once the capital, maintenance or wasted phosphorus fertilizer rates were determined for the appropriate crop, fertilizer rates were summed to the country and continent levels.

### Implications for phosphorus reserves

We calculated the impact of annual capital and maintenance fertilizer requirements and wasted phosphorus on the longevity of estimated phosphorus stocks. As of 2020, the global estimated stock of phosphate rock reserves amounted to 71,000,000 kt, or 9,301,310 kt of phosphorus after accounting for the P_2_O_5_ concentration in phosphate rock (30%) and the conversion of P_2_O_5_ into phosphorus^25^. We divided the estimated reserves (9,301,310 kt) by the annual fertilizer requirement for capital, maintenance and a third metric, termed optimal phosphorus, which accounted for the redistribution of wasted phosphorus. Optimal phosphorus was calculated as:

$${\mathrm{Optimal}}\,{\mathrm{phosphorus}}={\mathrm{Maintenance}}+\left({\mathrm{Capital}}-\left(\frac{{\mathrm{Wasted}}}{20\,{\mathrm{yr}}}\right)\right)$$

where maintenance included capital applications to new land to reach the threshold minus wasted phosphorus. We discounted wasted phosphorus by 20 years, which is the approximate time estimated for changing land management practices to be voluntarily implemented^65^. This accounts for the variable and slow redistribution of soil Olsen phosphorus from areas of excess to areas of deficit phosphorus with time.

### Reporting summary

Further information on research design is available in the Nature Portfolio Reporting Summary linked to this article.

### Supplementary information

Supplementary Information Supplementary Tables 1 and 2, Figs. 1–3 and text.

Reporting Summary

## Data Availability

All empirical data that support the main findings of this study have been deposited in figshare: 10.6084/m9.figshare.22137563.v1 (ref. ^66^). Publicly available datasets are available for cropland extent from https://lpdaac.usgs.gov/news/release-of-gfsad-30-meter-cropland-extent-products/ and land cover from https://www.esa.int/ESA_Multimedia/Images/2014/10/Land_cover_2010.
