# Supplementary material for: Phosphorus applications adjusted to optimal crop yields can help sustain global phosphorus reserves
Source: Nat Food. 2024 Mar 25;5(4):332–9. doi: 10.1038/s43016-024-00952-9 (PMC11045449; doi:10.1038/s43016-024-00952-9)
Supplement: Supplementary file 1 — Supplementary Tables 1 and 2, Figs. 1–3 and text. [file 43016_2024_952_MOESM1_ESM.pdf]

nature food

Analysis

https://doi.org/10.1038/s43016-024-00952-9

Phosphorus applications adjusted to optimal crop yields can help sustain global phosphorus reserves

In the format provided by the authors and unedited

Table of Contents

Data used to estimate plant Olsen phosphorus requirements 1
Model residuals.. 2
Wasted phosphorus by crop type 3
Validation of maintenance fertiliser applications.. 3
The effect of climate change on the phosphorus needs of maize and wheat 4

Data used to estimate plant Olsen phosphorus requirements

The data used to estimate the critical soil Olsen phosphorus concentrations for optimal yield were sourced for diverse locations from the literature (Supplementary Table 1). Optimal yield is defined as the 97th percentile of maximum relative yield derived from the fit of a Mitscherlich response curve to field data of crop growth against soil Olsen phosphorus concentration.

Supplementary Table 1. Critical soil Olsen phosphorus concentrations for the optimal yield of the most widely grown crop species globally.

Crop	Critical Olsen phosphorus concentration for optimal growth[1]	Countries where critical concentrations were established	Reference
Improved pasture[2]	15–25	Australia, New Zealand	1,2
Wheat	10–21#	Canada, USA, Europe, UK	3-7
Rice	11-17	Senegal, China	8,9
Maize	8–18	Ethiopia	10
	10	Tanzania	11
	8–12#	Nigeria	12
	18*	USA	3
	14	Europe	6
Soybean	11–17*	USA, Brazil	13,14
Barley	15	UK	7
Sorghum	6*	Benin, Burkina Faso, Ghana, Mali, Niger,	15
	9*	Europe, Nigeria, Mali	6,16
Millet	6*	Benin, Burkina Faso, Ghana, Mali, Niger, Nigeria	15
Cotton	3–6§	Australia, USA	17
Rapeseed	10	Australia	18
Groundnut	10	India	19
Sunflower	8–10	Australia	20
Sugarcane	12	Japan	21
Potato	20–33*	Europe, USA, Chile	6,22,23
Cassava	3–8	Colombia, USA	24,25
Oil palm	10	Sri Lanka	26
Rye	16–25	UK	27
Sugar beet	20–25	Europe, UK	6,28
Banana	10	USA	29
Cabbage	30–40	New Zealand, Norway	30,31
Rapeseed	10–20	Australia, Switzerland	18,32
Onion	43–60	New Zealand, Norway	30,31
Tomato	39–50	China, New Zealand	30,33
Grapes (wine)	7–10	USA	34,35
Watermelon	60	China	36
Cucumber	60	China	36
Orange	7–20	India	37,38
Apple	12–13	Canada, USA	39,40

[1] *, # and § = equivalent Olsen phosphorus concentrations calculated using the equations in Supplementary Table 3 for non-calcareous soils from Mehlich-3, Bray-I and Colwell-phosphorus data, respectively.

[2] Note that considerable variation in improved pasture yield is possible at low available soil phosphorus concentrations[41]. However, our data pertains to concentrations required for optimal yield of intensively grazed pastures, for example, for dairy production.

Model residuals.

Table S2. Mean percentage residuals between the observed and estimated (modelled) soil Olsen phosphorus concentrations for each continent split into classes. Data from McDowell, et al. [42]

Continent	0 to 2	>2 to 5	>5 to 10	>10 to 25	>25
Africa	-0.6	-1.2	0.9	3.3	26.1
Asia	-0.4	-1.9	-4.0	-6.8	63.8
Europe	-0.1	-0.8	-2.7	-9.0	0.4
North America	-0.2	-1.6	-4.1	-0.9	47.8
Oceania	-0.6	-2.3	-3.0	9.5	48.4
South America	-0.3	-1.3	-4.6	-4.3	51.4

Wasted phosphorus by crop type

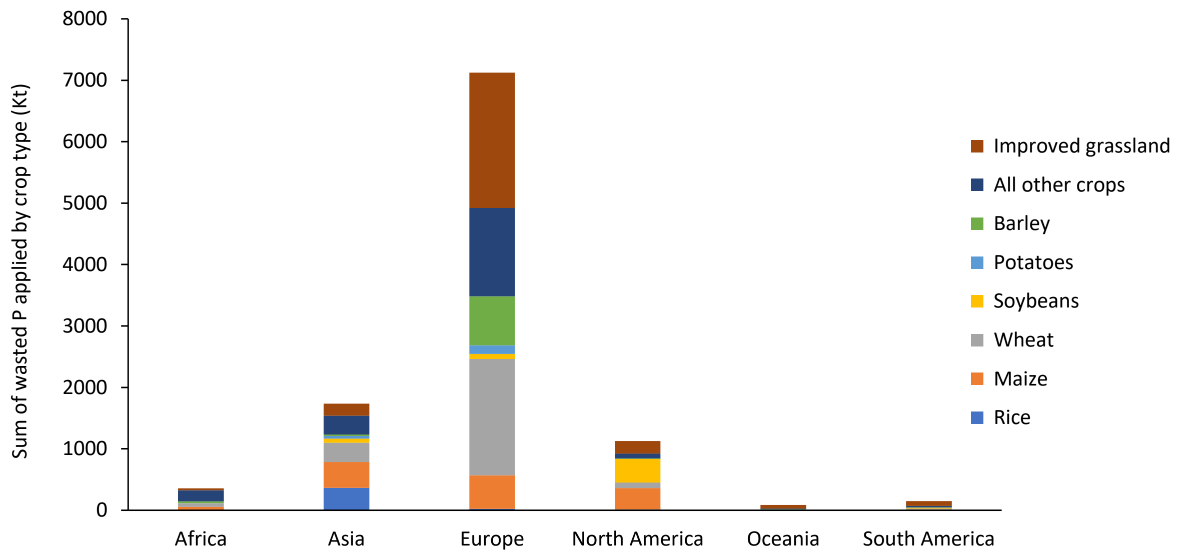

Supplementary Fig 1. Sum of wasted phosphorus (kt) applied to major crops grown in each continent.

Validation of maintenance fertiliser applications

Data were obtained for phosphorus fertiliser applications to Maize, Rice, Soybeans and Wheat in 24 countries (Argentina, Australia, Bangladesh, Belarus, Canada, Chile, China, Egypt, Indonesia, Iran, Japan, Malaysia, Mexico, Morocco, Pakistan, Philippines, Russia, South Africa, Thailand, Turkey, Ukraine, United States, Uzbekistan, and Vietnam) [43]. These represent the best freely available and audited data at the crop by country level and come from multiple sources as compiled by the International Fertilizer Association. We plotted these data against our estimates of the phosphorus required to maintain current soil Olsen phosphorus concentrations. Although dominated by two clusters of data, the regression was significant ($P<0.001$) and with a slope near to 1 (Supplementary Fig 2), suggesting that our estimates of maintenance phosphorus were reasonable. However, it is important to note that we could not validate wasted or deficit phosphorus nor phosphorus use in other countries.

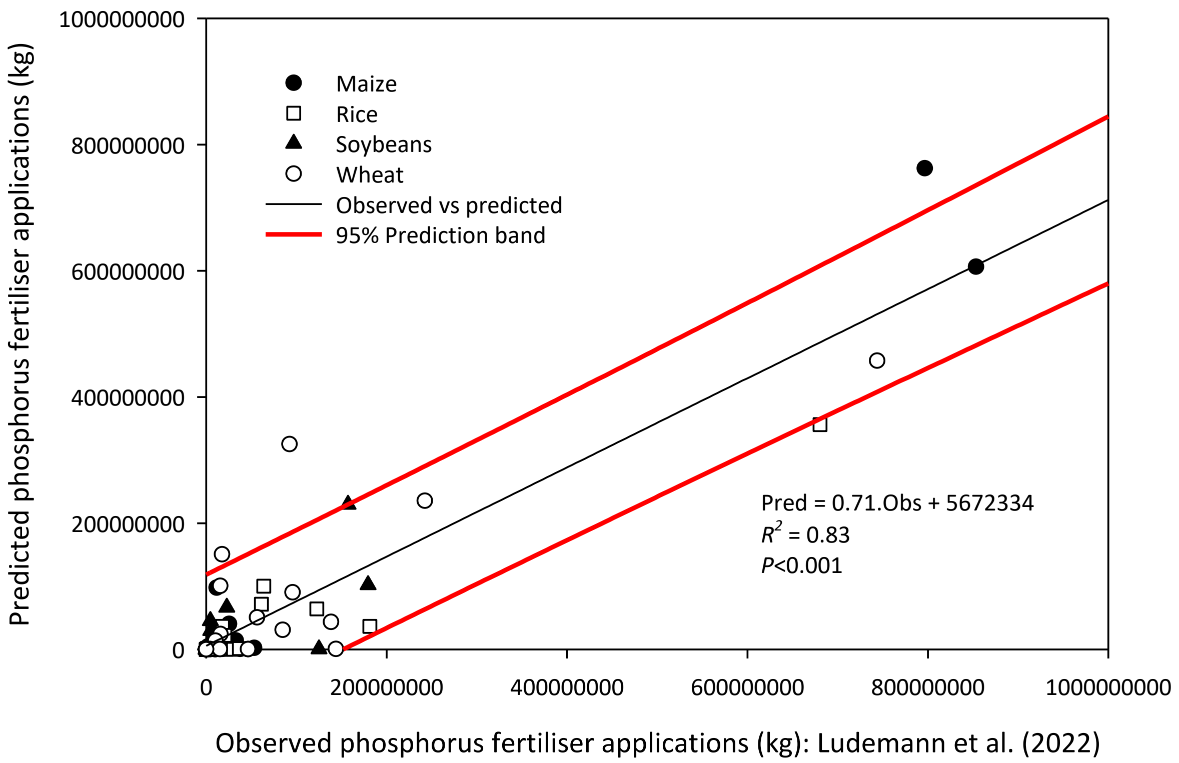

Supplementary Fig 2. Plot of predicted against observed fertiliser used across 24 countries for four crops. Observed fertiliser data were obtained from Ludemann, et al. [43]

The effect of climate change on the phosphorus needs of maize and wheat

Data were sourced from Jägermeyr, et al. [46] who produced predicted changes in the yield of maize and wheat in 2099 under the representative concentration pathways RCP2.6 and 8.5 across a range of climate and crop models for current growing regions (> 10 ha). We matched the predicted changes in percentage yield to Olsen P thresholds from existing relationships between Olsen P and relative yield [3-6,9,10,13]. For yield, decreases of -30, -20 and -10%, Olsen P was assumed to be the limiting factor and decreased from 15 to 8, 10 and 12 mg kg^{-1} respectively, with phosphorus requirements calculated according to soil phosphorus retention (assuming a phosphorus buffering index 2 – the most common soil type for these crops). For increases, soil phosphorus is unlikely to be limiting yield; hence, most fertiliser advice is to match phosphorus requirements with offtakes in grain [47,48] with residues being returned to the soil. For yield increases of 10, 20 and 30% we assumed that capital and maintenance Olsen phosphorus increased to 22, 28 and 35 mg kg^{-1}, respectively for maize and to 20, 25 and 30 mg kg^{-1}, respectively for wheat [48]. Too few data were available to calculate changes in rice and soybean. However, with changes likely to be only +/- 2% we assumed capital and maintenance requirements of +/- 1 mg kg^{-1} Olsen phosphorus, respectively.

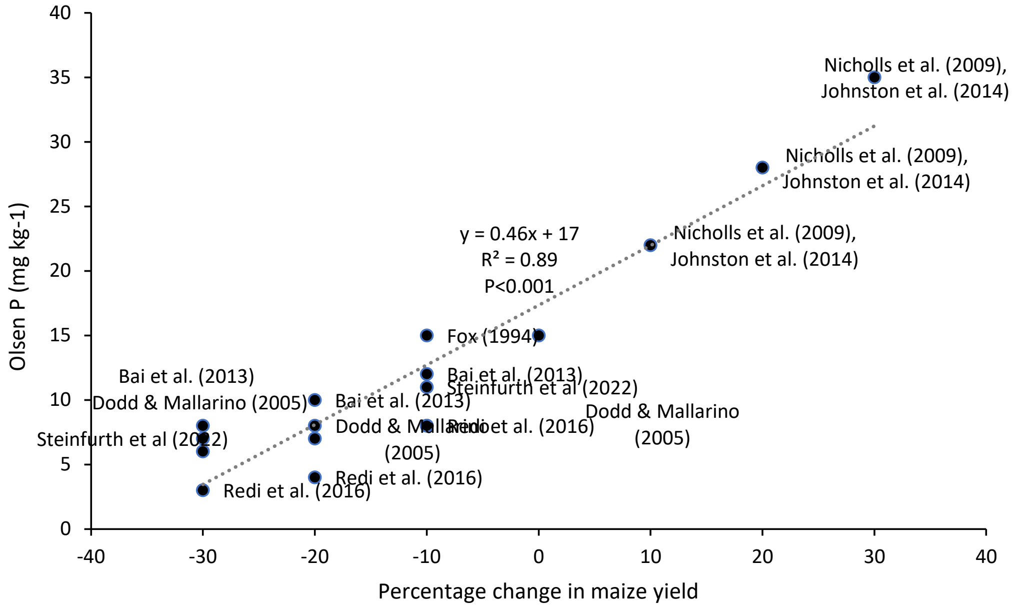

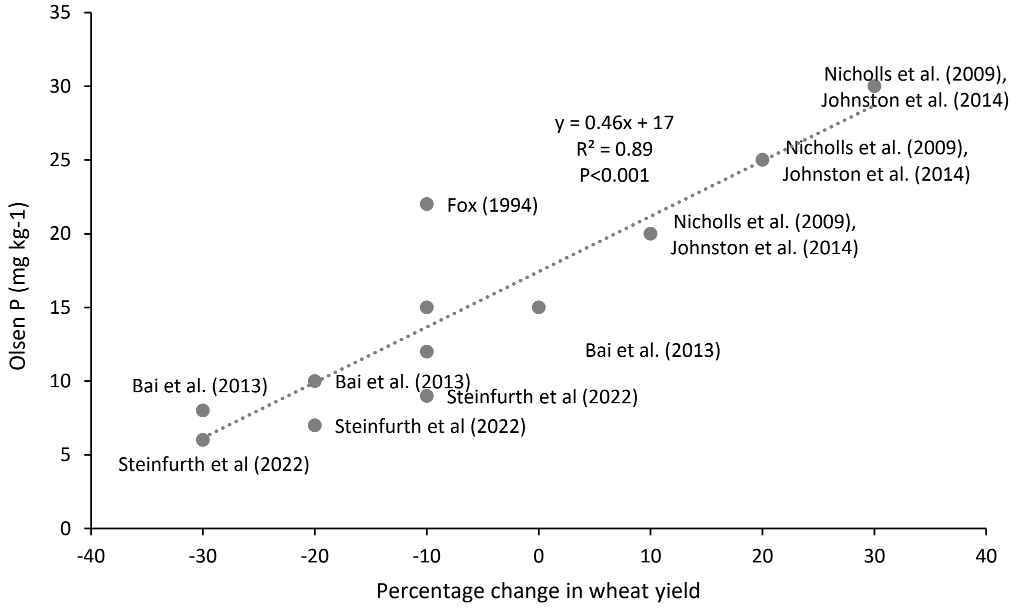

Supplementary Fig 3. Plot of the percentage change in maize or wheat yield against Olsen phosphorus concentration with labels indicating the source of the data.

References

1 Roberts, A. H. C. & Morton, J. D. Fertiliser use on New Zealand dairy farms. 52 (New Zealand Fertiliser Manufacturers' Research Association, Auckland, New Zealand, 2009).

2 Morton, J. D. & Roberts, A. H. C. *Fertiliser use on New Zealand sheep and beef farms*. 5th edn, (New Zealand Fertiliser Manufacturers' Research Association, 2016).

3 Cox, F. R. in *Soil testing: Prospects for improving nutrient recommendations* Vol. Special Publication 40 (eds J.L. Halvin & J.S. Jacobsen) 101-113 (SSSA, Madison, WI, 1994).

4 Read, D. W. L., Spratt, E. D., Bailey, L. D., Warder, F. G. & Ferguson, W. S. Residual value of phosphatic fertilizer on Chernozemic soils. *Can. J. Soil Sci.* **53**, 389-398 (1973).

5 Howard, A. E. Agronomic thresholds for soil phosphorus in Alberta: a review. 51 (Alberta Agriculture, Food and Rural Development, Saskatchewan, Canada, 2006).

6 Steinfurth, K. *et al.* Thresholds of target phosphorus fertility classes in European fertilizer recommendations in relation to critical soil test phosphorus values derived from the analysis of 55 European long-term field experiments. *Agric., Ecosyst. Environ.* **332**, 107926 (2022).

7 Syers, J. K., Johnson, A. E. & Curtin, D. Vol. FAO Fertilizer and Plant Nutrition Bulletin 18 (Food and Agriculture Organization of the United Nations, Rome, Italy, 2008).

8 Bado, B. V., De Vries, M. E., Haefele, S. M., Marco, M. C. S. & Ndiaye, M. K. Critical Limit of Extractable Phosphorous in a Gleysol for Rice Production in the Senegal River Valley of West Africa. *Commun. Soil Sci. Plant Anal.* **39**, 202-206 (2007).

9 Bai, Z. *et al.* The critical soil P levels for crop yield, soil fertility and environmental safety in different soil types. *Plant Soil* **372**, 27-37 (2013).

10 Redi, M., Gebremedhin, W., Merkeb, F. & YimamYimam, M. Critical Level of Extractable Phosphorus for Maize (Zea mays L.) at Metekel Zone, Northwestern Ethiopia. *World Scientific News* **54**, 14-26 (2016).

11 Ussiri, D. A., Mnkeni, P. N. S., MacKenzie, A. F. & Seraoka, J. M. R. Soil test calibration studies for formulation of phosphorus fertilizer recommendations for maize in Morogoro district, Tanzania. II. estimation of optimum fertilizer rates. *Commun. Soil Sci. Plant Anal.* **29**, 2815-2828 (1998).

12 Adeoye, G. O. & Agboola, A. A. Critical levels for soil pH, available P, K, Zn and Mn and maize ear-leaf content of P, Cu and Mn in sedimentary soils of South-Western Nigeria. *Fertilizer Research* **6**, 65-71 (1985).

13 Dodd, J. R. & Mallarino, A. P. Soil-Test Phosphorus and Crop Grain Yield Responses to Long-Term Phosphorus Fertilization for Corn-Soybean Rotations. *Soil Sci. Soc. Am. J.* **69**, 1118-1128 (2005).

14 Schlindwein, J. A., Bortolon, L., Fioreli-Pereira, E. C., Bortolon, E. S. O. & Gianello, C. Phosphorus and potassium fertilization in no till southern Brazilian soils. *Agricultural Sciences* **4**, 39-49 (2013).

15 Bationo, A., Kihara, J., Waswa, B., Ouattara, B. & Vanlauwe, B. in *Management of Tropical Sandy Soils for Sustainable Agriculture.* (eds C. Hatrmann *et al.*) (FAO Regional Office for Asia and the Pacific, 2005).

16 Doumbia, M. D., Hossner, L. R. & Onken, A. B. Variable sorghum growth in acid soils of subhumid West Africa. *Arid Soil Research and Rehabilitation* **7**, 335-346 (1993).

17 Dorahy, C. G., Rochester, I. J. & Blair, G. J. Response of field-grown cotton (Gossypium hirsutum L.) to phosphorus fertilisation on alkaline soils in eastern Australia. *Soil Res.* **42**, 913-920 (2004).
18 Brennan, R. F. & Bolland, M. D. A. Soil and Tissue Tests to Predict the Phosphorus Requirements of Canola in Southwestern Australia. *J. Plant Nutr.* **30**, 1767-1777 (2007).
19 Dhillon, N. S. & Sidhu, A. S. Evaluation of soil test methods of phosphorus for groundnut unde field conditions. *JJournal of the Indian Society of Soil Science* **40**, 478-482 (1992).
20 Lewis, D. C., Potter, T. D. & Weckert, S. E. The effect of nitrogen, phosphorus and potassium fertilizer applications on the seed yield of sunflower (Helianthus annuus L.) grown on sandy soils and the prediction of phosphorus and potassium responses by soil tests. *Fertilizer research* **28**, 185-190 (1991).
21 Matin, M. A., Oya, K., Shinjo, T. & Horiguchi, T. Phosphorus Nutrition of Sugarcane: Growth, Yield and Quality of Sugarcane as Affected by Soil Phosphorus Levels. *Japanese Journal of Tropical Agriculture* **41**, 52-59 (1997).
22 Rosen, C. J., Kelling, K. A., Stark, J. C. & Porter, G. A. Optimizing Phosphorus Fertilizer Management in Potato Production. *American Journal of Potato Research* **91**, 145-160 (2014).
23 Sandaña, P., Orena, S., Rojas, J. S., Kalazich, J. & Uribe, M. Critical value of soil Olsen-P for potato production systems in volcanic soils of Chile. *Journal of soil science and plant nutrition* **18**, 965-976 (2018).
24 Centro Internacional de Agricultura Tropical (CIAT). Cassava Program. Annual Report for 1982 and 1983. . (Centro Internacional de Agricultura Tropical (CIAT), Cali, Colombia).
25 Hue, N. V. & Fox, R. L. Predicting Plant Phosphorus Requirements for Hawaii Soils using a Combination of Phosphorus Sorption Isotherms and Chemical Extraction Methods. *Commun. Soil Sci. Plant Anal.* **41**, 133-143 (2010).
26 Pushparajah, E., Chan, F. & Magat, S. in *Phosphorus requirements for sustainable agriculture in Asia and Oceania* 399-408 (International Rice Research Institute, Manila, Phillippines, 1990).
27 Agriculture and Horticulture Development Board. Nutrient Management Guide (RB209), Section 4 Arable Crops. 52 (Agriculture and Horticulture Development Board, Kenilworth, Warwickshire, UK, 2020).
28 Johnston, A. E., Poulton, P. R. & White, R. P. Plant-available soil phosphorus. Part II: the response of arable crops to Olsen P on a sandy clay loam and a silty clay loam. *Soil Use. Manage.* **29**, 12-21 (2013).
29 Lin, M. L. *Phosphorus nutrition of Banana as influenced by mycorrhizae and fertilizers* PhD thesis, University of Hawaii, (1987).
30 Prasad, M., Spiers, T. M. & Ravenwood, I. C. Target phosphorus soil test values for vegetables. *New Zealand Journal of Experimental Agriculture* **16**, 83-90 (1988).
31 Uusitalo, R., Suojala-Ahlfors, T., Kivijärvi, P. & Hurme, T. Yield responses to P fertilisation of onion (Allium cepa L.) and cabbage (Brassica oleracea Capitata Group L.) in Finland. *Agricultural and Food Science* **27** (2018).
32 Cadot, S., Bélanger, G., Ziadi, N., Morel, C. & Sinaj, S. Critical plant and soil phosphorus for wheat, maize, and rapeseed after 44 years of P fertilization. *Nutrient Cycling in Agroecosystems* **112**, 417-433 (2018).

33 Zhang, X.-S. *et al.* Response of Tomato on Calcareous Soils to Different Seedbed Phosphorus Application Rates1 1Project supported by the National Natural Science Foundation of China (No. 30230250) and the Ministry of Agriculture, China (No. 2003-Z53). *Pedosphere* **17**, 70-76 (2007).
34 Moyer, M. M., Singer, S. D., Davenport, J. R. & Hoheisel, G.-A. Vineyard nutrient management in Washington state. 45 (Washington State University, Pullman, WA, 2018).
35 Grant, R. S. Managing phosphorus deficiency in vineyards. *Winegrowing* **January/February**, 87-90 (1999).
36 Chen, L. & Lu, J. *The vegetable nutrition and fertilization technology (In Chinese)* (China Agriculture Press, 2002).
37 Srivastava, A. K. & Patil, P. Nutrient Indexing in "'Kinnow" Mandarin (Citrus deliciosia Lour.× Citrus nobilis Tanaka) Grown in Indogangetic Plains. *Commun. Soil Sci. Plant Anal.* **47**, 2115-2125 (2016).
38 Lakshimi, L. M., Ramana, K. T. V., Patil, P. & Srivastava, A. K. Soil fertility norms for Sathgudi sweet orange (Citrus sinensis). *Indian J. Agric. Sci.* **87**, 1303-1306 (2017).
39 Raese, J. T. Phosphorus deficiency symptoms in leaves of apple and pear trees as influenced by available soil phosphorus. *Commun. Soil Sci. Plant Anal.* **33**, 461-477 (2002).
40 Munroe, J. *et al. Publication 611, Soil Fertility Handbook*. 3rd edn, 256 (Ontario Ministry of Agriculture, Food, and Rural Affairs, 2018).
41 Ros, M. B. H. *et al.* Towards optimal use of phosphorus fertiliser. *Scientific Reports* **10**, 17804 (2020).
42 McDowell, R. W., Noble, A., Pletnyakov, P. & Haygarth, P. M. A Global Database of Soil Plant Available Phosphorus. *Scientific Data* **10**, 125 (2023).
43 Ludemann, C. I., Gruere, A., Heffer, P. & Dobermann, A. Global data on fertilizer use by crop and by country. *Scientific Data* **9**, 501 (2022).
44 Ryan, J. *et al.* in *Advances in Agronomy* Vol. 114 (ed Donald L. Sparks) 91-153 (Academic Press, 2012).
45 Pritchett, W. L. & Gooding, J. W. Fertilizer recommendations for pines in the southeastern Coastal Plain of the Unites States. 23 (Gainsville, Florida, 1975).
46 Jägermeyr, J. *et al.* Climate impacts on global agriculture emerge earlier in new generation of climate and crop models. *Nature Food* **2**, 873-885 (2021).
47 Johnston, A. E., Poulton, P. R., Fixen, P. E. & Curtin, D. in *Advances in Agronomy* Vol. 123 (ed Donald L. Sparks) 177-228 (Academic Press, 2014).
48 Nicholls, A., van der Weerden, T. J., Morton, J. D., Metherell, A. & Sneath, G. *Managing soil fertility on cropping farms*. (New Zealand Fertiliser Manufacturers' Research Association, 2009).
